# Pose-Invariant Face Recognition via RGB-D Images

**DOI:** 10.1155/2016/3563758

**Published:** 2015-12-27

**Authors:** Gaoli Sang, Jing Li, Qijun Zhao

**Affiliations:** State Key Laboratory of Fundamental Science on Synthetic Vision, College of Computer Science, Sichuan University, Chengdu, Sichuan 610064, China

## Abstract

Three-dimensional (3D) face models can intrinsically handle large pose face recognition problem. In this paper, we propose a novel pose-invariant face recognition method via RGB-D images. By employing depth, our method is able to handle self-occlusion and deformation, both of which are challenging problems in two-dimensional (2D) face recognition. Texture images in the gallery can be rendered to the same view as the probe via depth. Meanwhile, depth is also used for similarity measure via frontalization and symmetric filling. Finally, both texture and depth contribute to the final identity estimation. Experiments on Bosphorus, CurtinFaces, Eurecom, and Kiwi databases demonstrate that the additional depth information has improved the performance of face recognition with large pose variations and under even more challenging conditions.

## 1. Introduction

Face recognition has been attracting considerable attention from researchers due to its wide variety of applications, such as homeland security, video surveillance, law enforcement, and identity management. The face recognition task can be performed nonintrusively, without user's awareness or explicit cooperation, thanks to the development of image sensing techniques. During the past decades, performance of face recognition is improving [1–5], but large pose variation problem still remains unsolved [6–8].

*2D Based Method.* Many researchers explored the large pose face recognition problem in recent years, and the popular 2D image based method achieved significant improvement. Deep learning based method has attracted researches' attention because of its boosted performance. Zhu et al. [4] learned face identity preserving features by using a deep neural network to transform a nonfrontal face to frontal. In [5], a new deep neural network named multiview perceptron (MVP) was designed to learn features with respect to identity and view using millions of face images. The features learned from MVP achieve satisfactory performance on the Multi-PIE dataset. However, both methods required an extremely large dataset to train. Our method, on the other hand, attempts to solve large pose variation problem using additional depth information. The complementary 3D data provides a different view to solve large pose variation problem.

*Passive 3D Based Method.* 3D face models can be achieved from 2D face images. Reference [9] reconstructed a 3D shape model from multiple 2D face images and generated a set of densely sampled 2D face images as templates for pose-invariant recognition. Reference [10] proposed a fully automatic system that matched the reconstructed faces under frontal view. It can handle continuous pose variation up to ±45 in yaw and ±30 in pitch angles. Yet face recognition under large pose variation is still challenging. One issue lies in the chicken and egg problem. On the one hand, accuracy of the reconstructed model depends on precise facial landmark localization. On the other hand, precise facial landmark still remains unsolved under large pose variations.

*Active 3D Based Method.* With the development of active 3D sensing techniques, it is possible to capture 3D face models without considering the pose. There are roughly two kinds of 3D sensors; one is depth cameras like Microsoft Kinect [11], affordable and fast, but is of low resolution, low precision, and low reliability. The other is 3D scanners like Minolta [12], producing high quality model but usually slow and expensive. Li et al. [11] utilized low-quality RGB-D data from a consumer level sensor to handle face recognition problem under various poses. Both texture image and depth maps were transformed to the frontal view for similarity calculation. However, symmetric filling on texture images involved in the frontalized face images often results in artifacts that may degrade the matching performance. Rather than frontalization on the 2D face image, [13] generated multiple face images under some predefined poses from 3D face models in the gallery, and the query face compared to all these projected images. Experiments show that recognition rate was higher than frontalization based method. Reference [14] proposed a resurfacing approach to tackle the challenge case with depth map corrupted by quantization noise, where the performance was highly dependent on precision of landmark localization and face alignment. Reference [15] used data acquired by high-quality 3D scanner and demonstrated that recognition rate is significantly higher by using both color and depth maps than that using depth alone. Reference [16] also combined color and depth for face recognition based on high-quality 3D face models captured using Inspeck Mega Capturor II 3D sensor. To deal with pose variation, the texture images are deformed according to rotation from depth maps and achieved 69.1% rank-1 recognition rate on Bosphorus database.

In this paper, we aim at solving large pose face recognition problem using RGB-D face images, by fusing both texture and depth into one framework via Joint Bayesian classifiers. Input of our algorithm is a RGB-D face image facing any direction (−90 degree to 90 degree), and output is identity of that person by similarity estimation between the input and that in the gallery.

The remainder of this paper is arranged as follows. The proposed method is shown in Section 2.  Section 3 presents experimental results and relevant discussions. And concluding remarks are drawn in the end.

## 2. Proposed Method

Suppose *Q* = {*I*, *D*} denotes the query face, where *I* is the input 2D face image from arbitrary view and *D* its corresponding depth map, with the assumption that *I* and *D* are semantically registered. Suppose *G* is the gallery set, where each identity corresponds to a RGB-D pair of the frontal view. In order to locate the most similar face, our face recognition system (1) calculates similarity between *Q* and each sample in *G*, and (2) the final recognition result is obtained using some pretrained classifiers. For similarity calculation, we separately estimate similarity values from texture images and depth maps. Nevertheless, different sources of similarity estimation should undergo the same preprocessing step: to register the query face *Q* to each of the samples in *G*.

**Figure 1:**
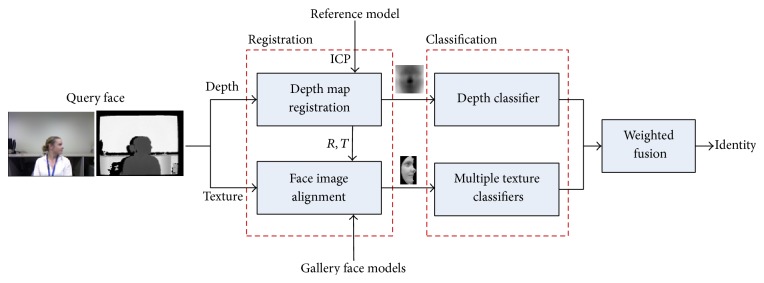
Framework of the proposed method. The input depth map *D* is first frontalized and registered to a predefined reference model. Its RGB counterpart *I* is then aligned to the database facing the same direction as the query. Finally, different classifiers are used for both texture and depth, and the final result is fused at score level. Here, ICP denotes the iterative closest points algorithm, and *R* and *T* are the rotation and translation matrixes estimated by ICP for registering the query face.

For depth map registration, we rotate the input profile face to the front via a 3D reference model *P*. And for texture image alignment, as its frontalized face is prone to artifacts, we on the other hand rotate the gallery to the input view. We then extract Histogram of Oriented Gradients (HOG) [18] features from the registered faces, which forms the basis for calculating similarity. For classification, we use different classifiers on texture and depth images separately. The final recognition result is obtained using some weighting scheme.  Figure 1 shows the framework of our algorithm.

### 2.1. Query-Gallery Face Registration

#### 2.1.1. Depth Map Registration

Given a query face *Q* = {*I*, *D*} facing any direction, we first estimate the pose-invariant frontal representation of the depth map, which can further be used for face recognition.

Several methods [19, 20] on face frontalization are discussed. In this paper, the frontalization task is solved by registering each input face to a reference model. Motivated by the method in [11], we build the reference model using standard face models with neutral expression from the USF [21] database. The reference face model is constructed by aligning all the faces in USF, resampling them on a uniform grid, and then taking their mean. The complete reference face has 128 × 128 points.

Suppose [*R*, *T*] represents rigid transformation between the query *Q* and reference model *P*, where *R* is the rotation matrix and *T* is translation vector. Thus, *Q* and *P* are registered via

(1) $$\begin{array}{c} P=RQ+T. \end{array}$$

We use iterative closest point (ICP) [22] to solve (1). ICP is an accurate technique for registration. To avoid its sensitivity to initial condition in classical ICP, here we corporate nose tip localization into ICP estimation. The nose tip positions are used for an initial and coarse matching between each query face and the reference face model. In this paper, we simply shift the original of the local coordinate system on the 3D face model to its nose tip position, so the two models can share the same coordinate system. At the end of the ICP registration, each query face and the reference model are registered. And we can obtain the pose normalized query face model and the corresponding rotation matrix *R* and transform vector *T*.

When the nose tip position is obtained, face cropping (both texture and depth) can also be easily done in 3D. The points that are more than 80 mm away from the nose tip are removed. Thus, the irrelevant data is abandoned. Note that the cropped faces are used in all our experiments.

After ICP registration, some data may be missing due to self-occlusion from nonfrontal query faces. Here, we estimate the values of the missing data through facial symmetry [11].

In order to reduce the noise and obtain a more reliable model, we smooth the point cloud (*XYZ*) using a publicly available code [23]. For each face, 128 × 128 points are normalized from its minimum *X* and *Y* to the maximum *X* and *Y* values.

#### 2.1.2. Texture Image Alignment

Reference [13] enlarged the gallery by rendering face images under different predefined poses and compared the query face with all templates in the gallery. However, density of the sampled poses matters. Too sparse sampling intervals will not help recognizing arbitrary view face images, whereas too dense sampling intervals will lead to high storage requirement. In this paper, we rotate each of the gallery face to the same view as the query and thus avoid facing the above dilemma.

2D face images are rendered from gallery faces facing the same direction as the probe by using pairs of rotation matrix *R* and transform vectors *T* obtained via ICP.

In our method, one frontal RGB-D face for each subject is needed in the gallery. Each of the samples in *G* can be transformed into *G*′ facing the same view as the probe by

(2) $$\begin{array}{c} G^{\prime }=R^{-1}G-R^{-1}\cdot T, \end{array}$$

where *R* ^−1^ denotes the inverse of the rotation matrix *R*.

We transform all of the face models in *G* through (2) and render texture images with the same view as the query image using weak perspective projection [24]. Similarity between query face and rendered one is then estimated accordingly.

So, once the rotation matrix *R* and transform vectors *T* of a query are determined via ICP obtained, the rendered face images are automatically registered from their corresponding 3D counterparts.

### 2.2. Classification

We employ HOG for both texture image and depth map feature extraction. HOG is proven to be robust against geometric and has been used successfully in many applications such as object detection and recognition. In general, we consider that the appearance and shape information of an image can be well described by gradient or the direction of the edge density distribution. HOG is defined as the histogram of image gradients over a combination of positions, orientations, and scales. Moreover, in order to improve performance when using HOG, the locality histogram is made to normalize the contrast in the larger scope of image.

Compared with other classifiers such as Support Vector Machine, neural network, *k*-Nearest Neighbor, and Classification Trees, Joint Bayesian [25] is easier to train and does not require retraining when new persons are enrolled.

In our proposed framework, we employ Joint Bayesian algorithm for face recognition. Specifically, Joint Bayesian classification is applied on both texture and depth maps. We design to learn multiple pose-specific classifiers for texture images, in order to handle various poses. Consider a continuous pose set ranging [−90,90] degree, each classifier is trained by a fixed degree partitioning (i.e., an interval of 10 degree). As for depth maps, all the frontal view depth maps in the training set are used to train a depth classifier. The match scores of texture and depth are fused via weighted summation as follows:

(3) $$\begin{array}{c} S=\lambda_{1}*S_{depth}+\lambda_{2}*S_{texture}, \end{array}$$

where *S*_depth_ and *S*_texture_ represent scores from depth maps and texture images, respectively. *λ*_1_ and *λ*_2_ are weighting parameters, which are determined based on the reliability of texture and depth. For instance, RGB-D images acquired by low-resolution sensors usually have less distinguishable depth maps than texture images, and hence the weight for depth score tends to be smaller than that for texture score. Finally, the query is assigned with the label of the class with highest similarity score.

## 3. Experiments

We test our method on four publicly available datasets, including (1) high-quality RGB-D data from Bosphorus database and (2) some more challenging datasets that contain low-quality depth capture and illumination changing along with various expressions (e.g., CurtinFaces) and occlusions (e.g., Eurecom and Kiwi). We compare our method with the prior-arts methods including PGM [16], PGDP [17, 26], DCT+SRC [11], COV+LBP [13], and RCRC [14]. We also evaluate in which way the 3D information can be fully explored and works better for 3D face recognition. Two experiments are designed; one is profile-to-profile depth comparison and the other is frontal-to-frontal mode. To further demonstrate robustness of our proposed method, we also investigate how the landmark location accuracy affects recognition rate of the proposed method.

Unless otherwise indicated, all the experiments HOG feature extraction was run on 9 × 9 patches and the gradient orientations between [0; 2*π* are evenly divided into 18 bins. As the texture and depth classifiers are designed separately, the final score is obtained by linearly fusing them using manually set weighting parameters 0.6 and 0.4, respectively.

According to our experimental results, ICP still works when out-of-plane rotation is 90 degrees. But we have to carefully choose the distance tolerance when establishing closest point correspondences. In our experiments, we empirically set it to 3 millimeters.

### 3.1. Evaluation on Bosphorus Dataset

The Bosphorus database contains 4666 textured depth maps of 105 persons in various poses, expressions, and occlusion conditions. The faces in the Bosphorus database are acquired using Inspeck Mega Capturor II 3D sensor that captures high-precision face models with accuracy of *x* = 0.3 mm, *y* = 0.3 mm, and *z* = 0.4 mm. For each 3D model, a subset of 24 facial landmarks (contains nose tip) is provided in the database. For each subject, there are various head poses including seven yaw angles, four pitch angles, and two cross rotations which incorporate both yaw and pitch. In order to ensure efficient and effective performance of the proposed method, a preprocessing step that downsample the high-precision face data is needed in our experiment.

**Figure 2:**
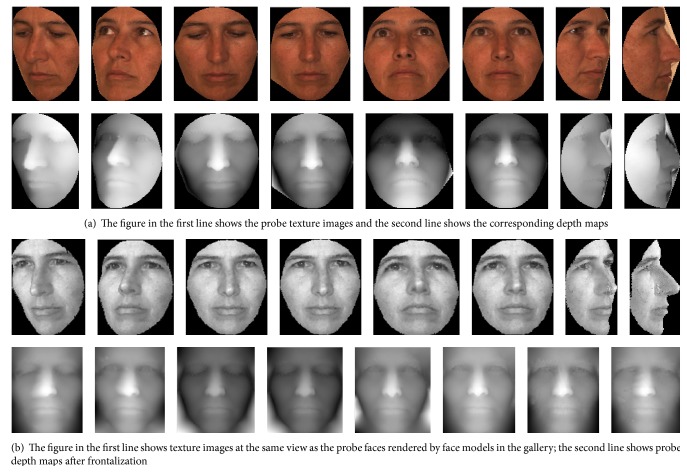
Samples of test images of one subject with poses variations.

We compare the performance of the proposed method with a set of approaches. PGM [16] transformed the texture map of a face model through its shape data via the Patch Geodesic Distance into a transformed texture map to tackle the missing data problem due to pose and expression changes. Both texture and spatial distance contribute to the final similarity measure between the query and the gallery face models. To handle the missing data problem caused by pose and expression changes, PGDP [17] transformed the texture map with pose variations through Patch Geodesic Distances into the view in-variance texture. Then shape adjusted texture patches are encoded into local patterns for face recognition. In our method, similarity measure on texture images is calculated among pairs of images that face the same direction as the query face. For each subject, one frontal neutral face model is used as the gallery set. All the frontal view face models for each subject are used to train 13 texture (including seven yaw angles, four pitch angles, and two cross rotations) and depth Joint Bayesian classifiers. 13 images with yaw, pitch, and cross rotation incorporate both yaw and pitch for each subject are used as the probe set.  Figure 2 shows the preprocessing results of some examples of a subject in the probe set. In this experiment, we set that *λ*_1_ equals *λ*_2_ (0.5) under the observation that depth maps are as distinguishable as texture images.

Table 1 summarizes the recognition rates on different methods with respect to different rotation angles on the Bosphorus database. Obviously, the proposed method is much better than other two methods. The proposed method achieves a 98.7% recognition rate for the +45° rotation to the right, while PGM and PGDP have the recognition rate of 39% and 36.2%. For the −45° rotation to the left, the recognition rate for the proposed method is 98.6% and PGM and PGDP are 38.1% and 37.1%, respectively. Again, for the right-downwards rotation, the recognition rate of the proposed method still outperforms PGM and PGDP by 58.2% and 57.3%, showing its consistent superior performance in accuracy. Moreover, For the +90°, −90° rotation, the proposed method achieves as much as 95.1% and 95.7% recognition rate, while PGM and PGDP have not reported.

The algorithm has been compared with other state-of-the-art methods testing on this database. Reference [26] transformed the query 3D face from many different poses to frontal pose by using the Hausdroff Distance metric and then extracted the corresponding normal values for face recognition. Reference [26] achieved the average rank-1 recognition rate that is 66%. It should be noted that [26] is based on 3D surface information, while the proposed method achieves an average accuracy of 87.1% on depth maps, outperforming the rank-1 recognition rate of [26] by 21.1%.

### 3.2. Evaluation on More Challenging Datasets

In this section, we evaluate our method using challenging data varying in quality, pose, expression, illumination, and occlusion conditions.

#### 3.2.1. Expression Variations on CurtinFaces Dataset

The CurtinFaces dataset contains more than 5000 images of 52 subjects with variations in poses, expressions, illumination, and sunglasses disguise acquired by Microsoft Kinect. Thus the face models in CurtinFaces are of low quality, and recognition using these data is challenging.

**Figure 3:**
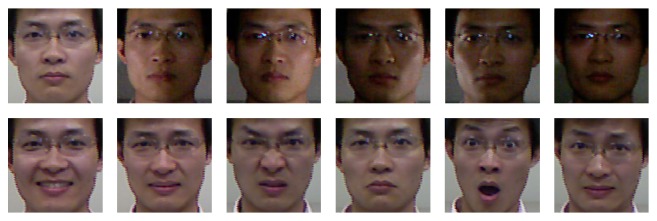
Samples of a subject used for training.

For a fair comparison with [11, 13, 14], we use the same subset that contains 7 poses (L90°, L60°, L30°, 0°, R30°, R60°, R90°) and 7 expressions (neutral, happy, disgust, anger, sad, surprise, and fear) per pose in the CurtinFaces database. In our experiment, 12 pairs of frontal views with various expressions and illuminations of each subject (see Figure 3) are used to train 9 texture classifiers (at about 0°, ±30°, ±60°, ±90° yaw rotation, and ±60° pitch rotation) and 1 depth classifier. 45 images variations in poses and expressions remaining per subject are used for testing. We compare the proposed method with the method in [13] which matches the texture maps with similar pose of the query face and the method in [11] matches the texture and the depth map with the gallery faces in a frontal view. For our method, one pair of RGB-D face under frontal view is used as the gallery for each subject. The recognition rate is reported in Table 2 for different poses under different expressions.

As shown in Table 2, we report the results achieved by texture, depth map, and fusion of texture and depth map using the proposed method. It is obvious that fusion of texture and depth information performs better than only using texture or depth map. Our method performs better with a certain degree of improvement, especially when the query faces at profile view, the recognition rates of texture and depth improve significantly compared with previous works. Exactly, compared with existing methods, the proposed method achieves 1.1% improvement than the second best method [14] at the profile view. Compared to the method in [13], the proposed method yields about 20.1% improvements at the profile view. No obvious drop in performance is observed across vast pose variations, even under the challenging case of profile view. Thanks to the ICP-based pose estimation method, our method projects gallery faces to the same view as the query, thus producing more accurate similarity measure. Moreover, our method aligns face images through 3D point clouds rather than several facial landmarks [14].

Furthermore, it can be observed from Tables 1 and 2 that the recognition rates for the depth only under the same view achieved by the Bosphorus database are higher than the results achieved under the corresponding head rotations by low quality data in the CurtinFaces dataset; the reason for this phenomena is that (1) in terms of depth precision high precision models provide more robust and accurate projection for texture rendering, thus producing more realistic rendered view compared with its low precision counterparts; (2) as in this paper, we did not handle nonrigid deformations (i.e., expression in CurtinFaces), and these data are treated as additional noise in our case; thus its recognition rate drops compare with that from noise-free data (i.e., Bosphorus).

#### 3.2.2. Occlusion Variations on Eurecom and Kiwi Databases

**Figure 4:**
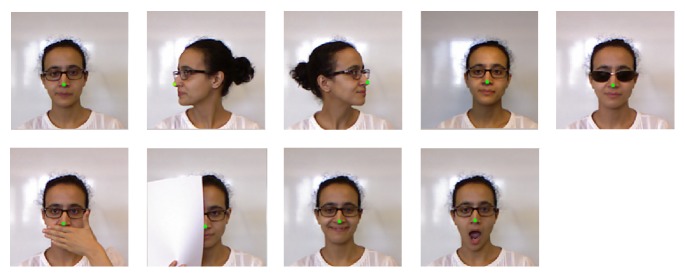
An example of a subject from Eurecom database with each nose tip is labelled. The rows from left to right are neutral, left profile and right profile, light on, and occlusion eyes; the bottom rows are occlusion mouth, occlusion paper, smile, and open mouth.

The Eurecom database contains 936 images of 52 subjects and the images are captured with variations in pose, illumination, and occlusion by Microsoft Kinect.  Figure 4 shows an example of 9 images of a subject in the Eurecom database. In this experiment, we use frontal faces except Occlusion Mouth and Occlusion Paper of each subject in Session 1 as training set to train three texture (frontal, left profile, and right profile) and one depth Joint Bayesian classifiers. The rest four images (left profile, right profile, occlusion mouth, occlusion paper) along with faces in Session 2 as the probe set.

**Figure 5:**
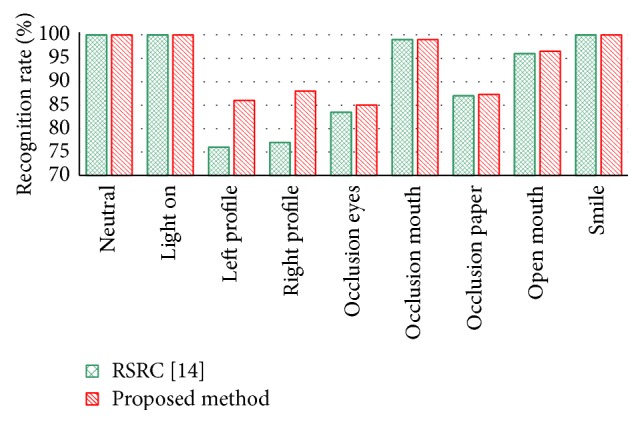
Performance comparison of the method RSRC by Hsu et al. and the proposed method on the Eurecom database with 9 conditions.

The recognition performance of the proposed method and the benchmark method are shown in Figure 5 under different conditions. By comparing the results of RSRC [14] and the proposed method with different conditions, it can be seen that the recognition rate of left and right profiles are significantly better than the benchmark method.

The Kiwi Kinect Head Pose database (abbreviated as Kiwi) contains over 15k images of 20 people (6 females and 14 males, 4 subjects recorded twice by with and without glasses) turning their head and captured at around 1 meter away from the sensor (Microsoft Kinect). The head pose range covers about ±90° yaw, ±60° pitch, and ±50° roll rotations. For a fair comparison with the methods RSRC [14], we use 10 pairs of frontal RGB-D faces of each subject to train 13 texture classifiers in which pose range covers about ±90° yaw rotations with an interval of 15 degree and one depth classifier.

**Figure 6:**
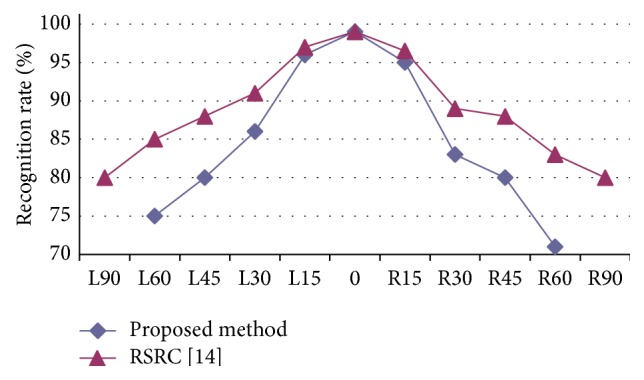
Performance comparison of different methods on the Kiwi database with different yaw angles ranging from −90 (L90) to +90 (R90) degrees.

The recognition performance of our method and RSRC are shown in Figure 6 with different yaw angles. The performances of the proposed method and RSRC are similar when the yaw angle ≤15°, although the former appears slightly better, while the recognition rate of RSRC degrades substantially when the yaw angle >15° because the performance of RSRC depends heavily on accuracy of facial landmark localization. However, facial landmark location is nontrivial for large pose face images due to self-occlusion.

### 3.3. Evaluation on Different Depth Comparison Mechanisms

In this part, we design experiments to explore the appropriate approach of 3D information that works better. We evaluate two different schemes for depth comparison. One approach is to rotate query face to the frontal view and then match to the gallery faces in a frontal view (denoted as frontal-to-frontal), while the other approach is to rotate each gallery face to the same pose as the query face; then the query face matches gallery face at nonfrontal view (denoted as profile-to-profile). For this comparison, we use a subset that contains only yaw pose variations of CurtinFaces database to evaluate the two approaches. One frontal face for each subject is used as the gallery set. Frontal faces with expression (seven different expressions) and illuminations (five different illuminations) of each subject are used as training set.

Interestingly, as shown in Table 3, the recognition rate achieved at frontal view is significantly better than nonfrontal view, especially under large pose variations. This is probably because the profile depth maps that are rotated from low-resolution frontal ones are seriously distorted. Furthermore, some information is lost due to self-occlusion when rotating. However, this is not the case for texture images, where profile-based comparison performs better, as the transformation from profile to frontal may produce artifacts, that is, illumination change.

### 3.4. Robustness on Nose Tip Localization

The proposed method required the nose tip for face cropping and rough alignment. In the above experiments, we suppose the nose tip is provided by database or marked manually. While in real-world applications, when using automatically algorithms to detect nose tip, there will be inevitably some errors in detecting nose tip locations. In order to evaluate the impact of nose tip location on face recognition accuracy, we perform proposed method using noisy location of nose tip on the CurtinFaces database. Specifically, we disturb the ground truth nose tip by randomly selecting the nose tip within a radius of 3, 5, 7, and 9 mm of the ground truth location. We conduct two series of experiments on the subset that contains only yaw pose variations of CurtinFaces database: (1) training and testing both using nose tip provided by database or manually marked (denoted as groundtruth data); (2) training using nose tip provided by database or manually marked and testing using data with noisy nose tip location (denoted as noisy data).  Table 4 presents the recognition rates of the proposed method testing with ground truth and noisy nose tip location.

As shown by Table 4, our proposed method is not sensitive to the noisy nose tip location. The larger the radius we test is, the stronger the noise is. As can be seen, the recognition rate is comparably the same as intensity of the noise growth. When the radius is as large as 9 mm, the recognition rates only decline about 0.9%. The results prove that our proposed method can perform well even when the nose tip location is not as accurate.

### 3.5. Time Complexity

Table 5 lists the average training and test time of our proposed method using a 64-bit MATLAB implementation on a PC with Intel i3 CPU and 4 GB memory. No extra effort was made for code optimization. In the training phase, most of the time is due to the 2D face rendering process. In other words, rendering 2D face images from each of the gallery faces to the same direction as the probe is the most time-consuming step in the proposed method.

In terms of computational complexity, the proposed method takes more time than the DCT+SRC method to recognize a single query face mainly due to 2D face rendering process. On average, the computational complexity of our algorithm is 9 s for recognizing a single query face from a database of 52 persons, while the time consumption on verification is only about 4.5 s. If the proposed method could be implemented using C/C++ with code optimization and parallel computation, we expect an improvement over its efficiency.

## 4. Conclusions

In this paper, we presented a pose-invariant face recognition method using depth and texture. We evaluated our proposed method on four public available face databases, and the results demonstrated that the proposed method can not only handle large pose variation problem, but also achieve promising results under challenge conditions like expression and occlusion.

A limit of the proposed method is its high computational cost when identifying a person in a large gallery. In this sense, it is more applicable for face verification (i.e., one-to-one matching). In our future work, we are going to investigate the possibility of speeding it up via parallel computing. In addition, we are also going to exploit other feature representation methods to further improve the proposed method.

## Figures and Tables

**Table 1:** Recognition rates for different pose rotations on the Bosphorus database.

Pose	PGM [16]	PGDP [17]	Proposed method		
			Depth	Texture	Fusion
+10° right	92.3%	96.1%	90.0%	100%	99.9%
+20° right	88.6%	91.4%	89.5%	99.7%	99.9%
+30° right	80.0%	80.1%	89.3%	99.6%	99.8%
+45° right	39.0%	36.2%	88.8%	97.7%	98.7%
−45° left	38.1%	37.1%	88.6%	98.1%	98.6%
+90° right	N/A	N/A	84.2%	91.4%	95.1%
−90° left	N/A	N/A	84.5%	92.3%	95.7%
Upwards (slight)	87.6%	96.2%	88.9%	93.4%	97.3%
Upwards (strong)	79.0%	91.4%	87.1%	92.0%	96.6%
Downwards (slight)	87.6%	86.7%	87.1%	93.0%	97.8%
Downwards (strong)	69.5%	70.0%	85.4%	92.7%	94.8%
Right-upwards	63.8%	64.7%	85.6%	91.2%	95.6%
Right-downwards	34.3%	35.2%	84.3%	89.0%	92.5%
Average	69.1%	71.4%	87.1%	93.4%	96.7%

N/A denotes that the measure is not available in the paper where the method was proposed (the same for the other tables in this paper).

**Table 2:** Recognition rates of different methods on the pose-expression subset of the CurtinFaces database.

Pose	COV+LBP [13]	DCT+SRC [11]			RSRC [14]	Proposed method		
		Depth	Texture	Fusion		Depth	Texture	Fusion
Frontal	N/A	100%	100%	100%	100%	100%	100%	100%
±30° yaw	94.2%	88.3%	99.8%	99.4%	99.4%	88.5%	99.5%	99.5%
±60° yaw	84.6%	87.0%	97.4%	98.2%	98.2%	87.7%	97.5%	98.4%
±90° yaw	75.0%	74.0%	83.7%	84.6%	93.5%	78.5%	94.0%	95.1%
±60° pitch	N/A	81.6%	89.1%	92.8%	N/A	82.5%	94.3%	96.7%

For COV+LBP, a subset of pose-variation of the CurtinFaces is used.

For RSRC, we report the average of the best results at the same yaw pose obtained by the method.

**Table 3:** Recognition rates when two different schemes are used for head rotations of depth map.

Pose	Depth map	
	Frontal-to-frontal	Profile-to-profile
±30° yaw	89.4%	86.5%
±60° yaw	87.5%	72.1%
±90° yaw	78.8%	57.6%

**Table 4:** Recognition rates testing with groundtruth and noisy nose tip location.

Pose	Groundtruth data	Noisy data			
		*R* = 3 mm	*R* = 5 mm	*R* = 7 mm	*R* = 9 mm
±30° yaw	99.1%	98.1%	99.1%	99.1%	98.1%
±60° yaw	98.1%	98.1%	98.2%	97.2%	97.2%
±90° yaw	95.2%	95.2%	95.2%	94.2%	94.2%

**Table 5:** Time performance comparison of the proposed and DCT+SRC methods on the CurtinFaces database (average time in seconds).

	Training (ours)	Testing	
		Single query [11]	Single query (ours)
Face cropping	89	0.061	0.127
ICP registration	3230	3.467	3.459
Symmetric filling	917	0.989	0.941
Resampling	174	0.477	0.002
2D face rendering	37440	—	4.024
Classification (depth)	13	0.026	0.036
Classification (texture)	15	0.084	0.057
Fusion	—	0.017	0.024

Total	44562	5.114	8.748
